# Common variants near *IKZF1* are associated with primary Sjögren's syndrome in Han Chinese

**DOI:** 10.1371/journal.pone.0177320

**Published:** 2017-05-26

**Authors:** Susu Qu, Yang Du, Suhua Chang, Liyuan Guo, Kechi Fang, Yongzhe Li, Fengchun Zhang, Kunlin Zhang, Jing Wang

**Affiliations:** 1 CAS Key Laboratory of Mental Health, Institute of Psychology, Beijing, China; 2 Department of Psychology, University of Chinese Academy of Sciences, Beijing, China; 3 Department of Rheumatology and Clinical Immunology, Peking Union Medical College Hospital, Peking Union Medical College and Chinese Academy of Medical Sciences, Key Laboratory of Rheumatology and Clinical Immunology, Ministry of Education, Beijing, China; National Cancer Institute, UNITED STATES

## Abstract

Primary Sjögren's syndrome (pSS) is a systematic autoimmune disease with evidence of genetic predisposition. The *IKZF1* (IKAROS family zinc finger 1 (Ikaros)) gene is located at 7p12.2, encodes a transcription factor related to chromatin remodeling, regulates lymphocyte differentiation, and has been reported to be associated with some autoimmune diseases. However, there have been no reports of an association between *IKZF1* and pSS. To investigate the possibility of an association between the *IKZF1* locus and pSS, we selected two single nucleotide polymorphisms (SNPs) in the *IKZF1* locus, rs4917129 and rs4917014, based on a detailed analysis of genome-wide association study (GWAS) data and performed genotyping in 665 Han Chinese pSS patients and 863 healthy controls. The results of an association test showed significant association signals (rs4917129: *P*-value = 5.5e-4, OR (odds ratio) = 0.72, 95% CI (confidence interval) = 0.60–0.87; rs4917014: *P*-value = 1.2e-3, OR = 0.76, 95% CI = 0.64–0.89). A meta-analysis that combined the above results with data from previous GWAS, further confirmed these associations (rs4917129: *P*_meta_ = 4.24e-8, OR_meta_ = 0.70, 95% CI = 0.61–0.79; rs4917014: *P*_meta_ = 6.0e-8, OR_meta_ = 0.72, 95% CI = 0.64–0.81). A bioinformatics analysis indicated that both SNPs were located in a putative enhancer area in immune-related cell lines and tissues. A protein-protein interaction analysis found that *IKZF1*, together with *GTF2I* (an SS susceptibility gene newly identified through GWAS), could interact with histone deacetylase family proteins. In summary, this is the first study to report an association between *IKZF1* and SS in Han Chinese.

## Introduction

Sjögren's syndrome (SS), a systemic autoimmune disease that involves the exocrine glands and is characterized by epithelial lymphocytic lesions at multiple sites, can also simultaneously affect other organs (e.g., lung, kidney, or liver). SS may occur alone (primary SS, pSS) or in association with other autoimmune diseases (secondary SS)[1]. pSS is one of the most common autoimmune diseases and affects approximately 0.05–4.8% of the global population[2]. The pathogenesis of pSS is still unknown, but there is evidence that a complex interaction between genes and environmental factors is involved in its etiology. In recent years, numerous genetic studies have been conducted to identify susceptibility genes for complex diseases[3, 4]. Among these, genome-wide association studies (GWAS) have dramatically promoted the progress of this research by screening for potential common variants associated with diseases using genome-wide high-throughput genotyping arrays. Currently, two pSS GWAS[5, 6] have been published in European and Chinese populations respectively, but they identified only a limited number of genome-wide significant susceptibility genes. The genetic factors contributing to the pathogenesis of pSS are still unclear, leaving an open area rich for exploration.

The *IKZF1* (IKAROS family zinc finger 1) gene is located at 7p12.2 and encodes a lymphoid-restricted zinc finger transcription factor that regulates lymphocyte differentiation and proliferation as well as self-tolerance through the regulation of B-cell receptor signaling. This gene was previously revealed to be involved in the regulation of *STAT4* in human T cells[7]. Previous studies also found that *IKZF1* is significantly associated with several autoimmune diseases, such as systemic lupus erythematosus (SLE)[8], Crohn's disease[9] and inflammatory bowel disease (IBD)[10]. However, it is unclear whether variants of this gene confer protection against pSS.

To our knowledge, there is no information in the literature about *IKZF1* polymorphisms and their relationship to pSS. The aim of the current study was to explore the association between common variants (single nucleotide polymorphisms, SNPs) near the *IKZF1* locus and pSS risk in an independent Han Chinese sample. Based on previous GWAS and imputation data, we developed a reasonable strategy for selecting SNPs (see the Methods section). We first genotyped two SNPs (rs4917129 and rs4917014) near the *IKZF1* locus in a Chinese Han population with 665 cases and 863 healthy controls and performed an association test. Then, we conducted a meta-analysis by combining our results with data from previous GWAS. Finally, we performed a bioinformatics analysis to explore the possible functions of the variants and this gene.

## Materials and methods

### Study samples

The present study included 665 pSS cases (mean age 49.32±12.74 years) consisting of 642 females and 23 males who were recruited with the cooperation of 40 centers in China. All the cases were diagnosed with pSS by at least two rheumatologists according to the American-European Consensus Group (AECG) criteria for pSS[11]. They were not diagnosed with any other autoimmune diseases at the same time. The control group included 863 healthy subjects (mean age 41.75±12.51 years), consisting of 840 female and 23 male adults. All the controls were recruited from Peking Union Medical College Hospital[5]. Our study was approved by the appropriate institutional review board of Peking Union Medical College Hospital, and appropriate written informed consent was obtained from all participants at recruitment. For all the case and control individuals recruited in our study, anti-Ro (SSA) and anti-La (SSB) antibodies were detected using ELISA (EUROIMMUN AG, Luebeck, Schleswig-Holstein, Germany).

### Imputation and regional association plot

To select suggestive signals around *IKZF1* for further validation in an independent Han Chinese population based on previously published GWAS data[5], we imputed the non-genotyped SNPs of the suggestive associated region around *IKZF1* (49.30–51.32 M of chromosome 7; positions were based on human genome version 19 (hg 19)) using MACH 1.0[12] with a reference panel from the CHB (Northern Han Chinese) data (103 individuals) of the 1000 Genomes Project Integrated Phase 3 (Phase3_shapeit2_mvncall_integrated_v5a.20130502). The quality of imputation was determined by calculating the squared correlation between imputed and true genotypes (Rsq), and only imputed SNPs with Rsq > 0.5 were retained for further analysis. Regional association results were plotted with LocusZoom[13].

### SNP selection and genotyping

A three-step strategy was used to select SNPs: first, imputation was performed to collect more information around *IKZF1* according to the GWAS data, and SNPs with *P*-values < 1E-3 were retained. Then, a conditional logistic regression analysis was performed using the top significant SNPs to detect independent signals at this locus. Finally, the top SNPs representing independent signals were selected. Specifically, after the first step, the only SNPs that showed significant association signals (*P*-values < 1E-3) and had been genotyped in previous GWAS were rs4917129 and rs4917014. The results of the conditional logistic regression analysis showed that one association signal could be indexed by either rs4917129 or rs4917014. Thus, both rs4917129 and rs4917014 were selected. For the genotyping procedure, we first extracted genomic DNA from peripheral blood samples from each individual using a Whole-Blood DNA Extraction Kit (Solution Type) (BioTeke). Then, genotyping was performed using the iPLEX MassARRAY platform (Sequenom). For quality control, we checked the SNP call rate (> 95%), Hardy-Weinberg equilibrium (HWE) for controls (*P*-values > 1E-3), minor allele frequency (>0.05) and call rate difference between cases and controls (*P*-values > 1E-5).

### Statistical analysis

The HWE test for genotypic distributions of controls was assessed with a χ^2^ test for goodness of fit. Comparisons of genotype and allele frequencies between the two groups were performed for each polymorphism with PLINK v1.07[14] using an additive logistic regression model (adjusting for sex and age), including calculations of the corresponding odds ratios (ORs) and 95% confidence intervals (CIs). To combine results from previous GWAS, a meta-analysis was conducted with a fixed-effects model using PLINK. Linkage disequilibrium (LD) statistics (D’ and *r*^2^) were calculated using Haploview 4.2 software[15]. A haplotype-based association analysis using logistic regression between these two SNPs was conducted using PLINK (adjusting for age and sex). *P*-values in all the association tests were two-sided.

### Functional prediction of significant SNPs

We performed a bioinformatics analysis to predict the potential functional consequences of the significant SNPs using rVarBase[16] (http://rv.psych.ac.cn/) and RegulomeDB[17] (http://www.regulomedb.org/), both of which annotate SNPs with multiple types of functional data (e.g., transcription factor-binding data, epigenetic sequencing information and eQTLs).

### Protein-protein interaction analysis

Numerous studies have shown that disease risk genes tend to function together through protein-protein interaction (PPI) networks[18, 19]. To further investigate the relationship between associated genes and pSS risk, we performed a PPI analysis for *IKZF1* and related genes. We first downloaded well-defined PPI data from Inweb[20, 21] and then searched for genes that physically interact with *IKZF1*. To investigate whether *IKZF1* physically interacts with proteins encoded by other pSS-associated genes, we then carefully curated high-confidence pSS susceptibility genes from published GWAS and candidate gene studies, and we extended the PPI network by searching for proteins that interact with pSS susceptibility genes.

## Results

**Fig 1:**
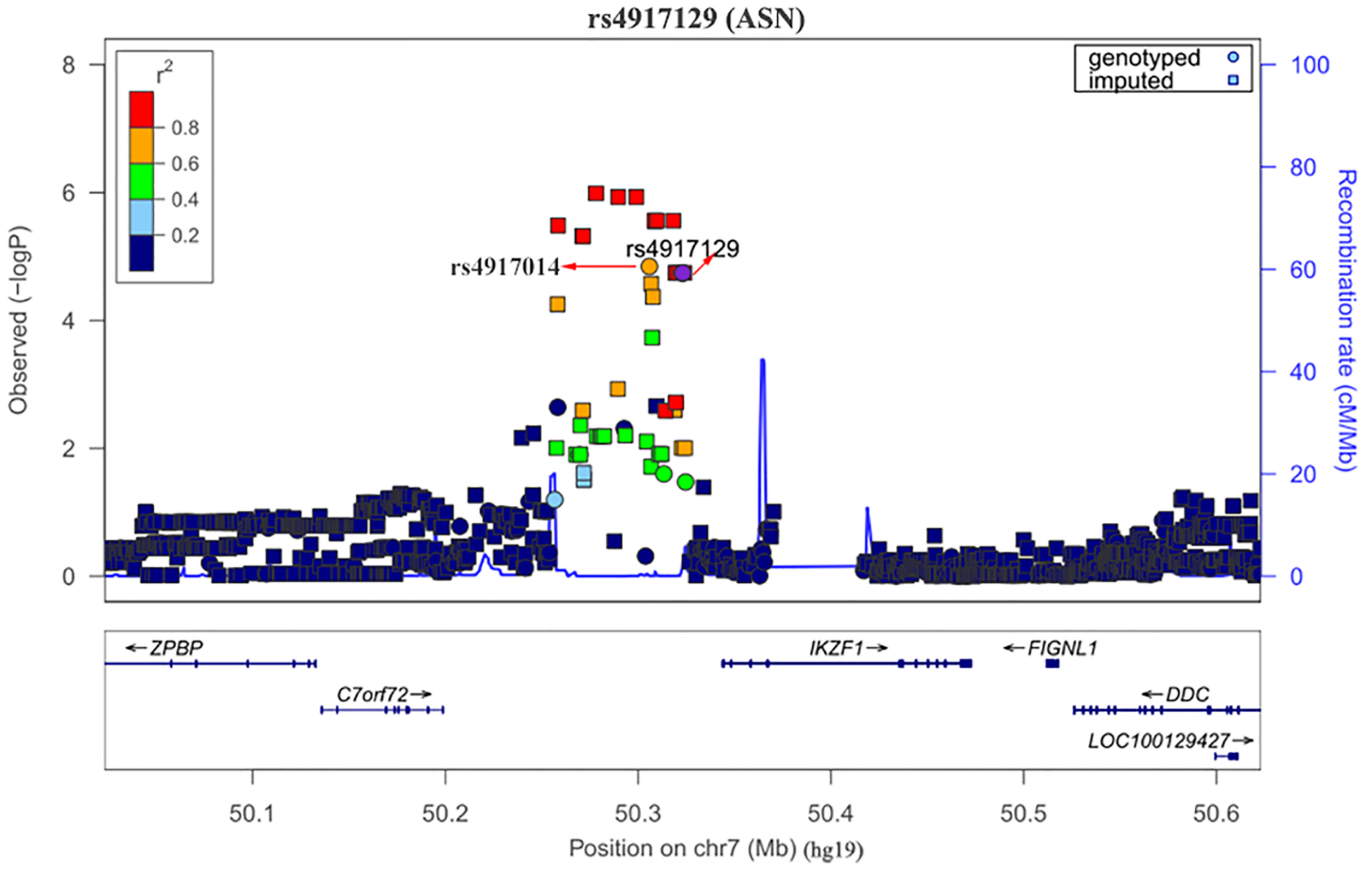
Regional association plot results for the primary Sjögren’s syndrome susceptibility locus near *IKZF1*. The plot shows the association results for both the genotyped (diamonds) and imputed (circles) SNPs in the GWAS samples. The -log10 *P*-values (left y-axis) of the SNPs are presented according to their chromosomal positions (x-axis). Genetic recombination rates, which were estimated using all the HapMap2 Project samples, are represented by light-blue lines, and genes within the regions are shown in the bottom panel. SNP rs4917129 is depicted as the larger purple circle labeled with the rs number, and its LD (*r*^2^ based on ASN data from the 1000 Genomes Project, November 2014) with the remaining SNPs is characterized by different colors. The position of rs4917014 is also labeled in the figure.

We performed imputation to further explore the details of association signals. The results showed that 4133 SNPs were imputed with high quality (Rsq > 0.5) at the *IKZF1* locus, and 428 SNPs were genotyped in the same area. The imputed SNPs with high quality, *P*-values < 1E-3 and near the *IKZF1* locus are shown in S1 Table. We found many SNPs upstream of the *IKZF1* gene that reached the level of significance (*P*-values < 1E-3), but only two of these SNPs (rs4917014 and rs4917129) were genotyped in our previous study. A conditional analysis revealed that either of these two SNPs might represent the sole signal in this region (for details, see S1 Table), and there was no residual association between these two SNPs in this region. The regional association plot for this susceptibility locus is shown in Fig 1. Then, we genotyped these two SNPs in 1528 subjects (including 665 cases and 863 healthy controls). A brief description of the subjects' features is provided in Table 1. There was no significant difference in the sex distribution of the subjects between the cases and controls. rs4917014 and rs4917129 were successfully genotyped in 99.9% of the subjects, and the genotype distributions in the control group were consistent with HWE (*P*-value = 0.69 and 0.72 for rs4917014 and rs4917129, respectively).

**Table 1:** Summary of subjects in our study.

		Total	Case	Control	*P*-value
**N**		1528	665	863	-
**Female (%)**		1482(0.970)	642(0.965)	840(0.973)	0.2^‡^
**Age (years)**^a^	**Range**		14–88	19–87	-
	**Mean**±**SD**		49.32±12.74	41.75±12.51	<0.001*

^‡^Pearson’s χ^2^ test.

*T-test

^a^All ORs and *P*-values were adjusted by age and sex.

We evaluated the associations between the SNPs and pSS risk using an additive logistic regression model (*P*-values were calculated with sex and age as covariates), and both rs4917014 and rs4917129 showed a significant association with pSS (rs4917129, *P*-value = 5.5e-4, OR = 0.72, 95% CI = 0.60–0.87; rs4917014, *P*-value = 1.2e-3, OR = 0.76, 95% CI = 0.64–0.89; see Table 2). The minor alleles of both SNPs exhibited a protectived effect against pSS and showed good agreement with previous GWAS. To achieve greater statistical power, we further combined these results with those of previous GWAS using a meta-analysis. The results showed that rs4917129 was associated with pSS with genome-wide significance (*P*_meta_ = 4.2e-8, OR_meta_ = 0.70, 95% CI = 0.61–0.79). rs4917014 also showed a highly significant association with pSS (*P*_meta_ = 6.0e-8, OR_meta_ = 0.72, 95% CI = 0.64–0.81). The levels of LD between the two SNPs in our previous GWAS control sample and replication control sample were both consistent with that of the 1000 Genomes CHB sample (previous GWAS: *r*^2^ = 0.60, D’ = 0.89; replication sample: *r*^2^ = 0.61, D’ = 0.90; 1000 Genomes CHB: *r*^2^ = 0.670, D’ = 0.91). Moreover, the haplotype analysis revealed two significant haplotypes (rs4917014-rs4917129: G-C and T-T) in our replication samples (*P*-value = 0.000143 and *P*-value = 0.0029, respectively), consistent with the results of previous GWAS samples (*P*_(G-C)_ = 7.65e-07 and *P*_(T-T)_ = 6.73e-05).

**Table 2:** Summary of association results for two SNPs associated with primary Sjögren's syndrome in Han Chinese.

SNP	Location^a^	Allele^b^	Gene	Dataset	MAF in cases	MAF in controls	OR^c^ (95% CI)	*P*-value^c^
rs4917129	7:50323174	C/T	*IKZF1*	Current study	0.2075	0.2509	0.72(0.60,0.87)	5.49×10^−4^
				Pre-GWAS^d^	0.191	0.2595	0.68(0.56,0.81)	1.81×10^−5^
				Combined	0.2001	0.2556	0.70(0.61,0.79)	4.24×10^−8^
rs4917014	7:50305863	G/T	*IKZF1*	Current study	0.2609	0.3045	0.76(0.64,0.89)	1.15×10^−3^
				Pre-GWAS^d^	0.2431	0.3161	0.68(0.57,0.81)	9.86×10^−6^
				Combined	0.2529	0.3109	0.72(0.64,0.81)	6.02×10^−8^

Chr., chromosome. MAF, minor allele frequency.

^a^Positions are based on human genome version 19 (hg 19).

^b^Minor/major alleles.

^c^ORs and *P*-values were calculated with an additive genetic model and were adjusted for age and sex.

^d^Pre-GWAS indicates previous GWAS results.

**Fig 2:**
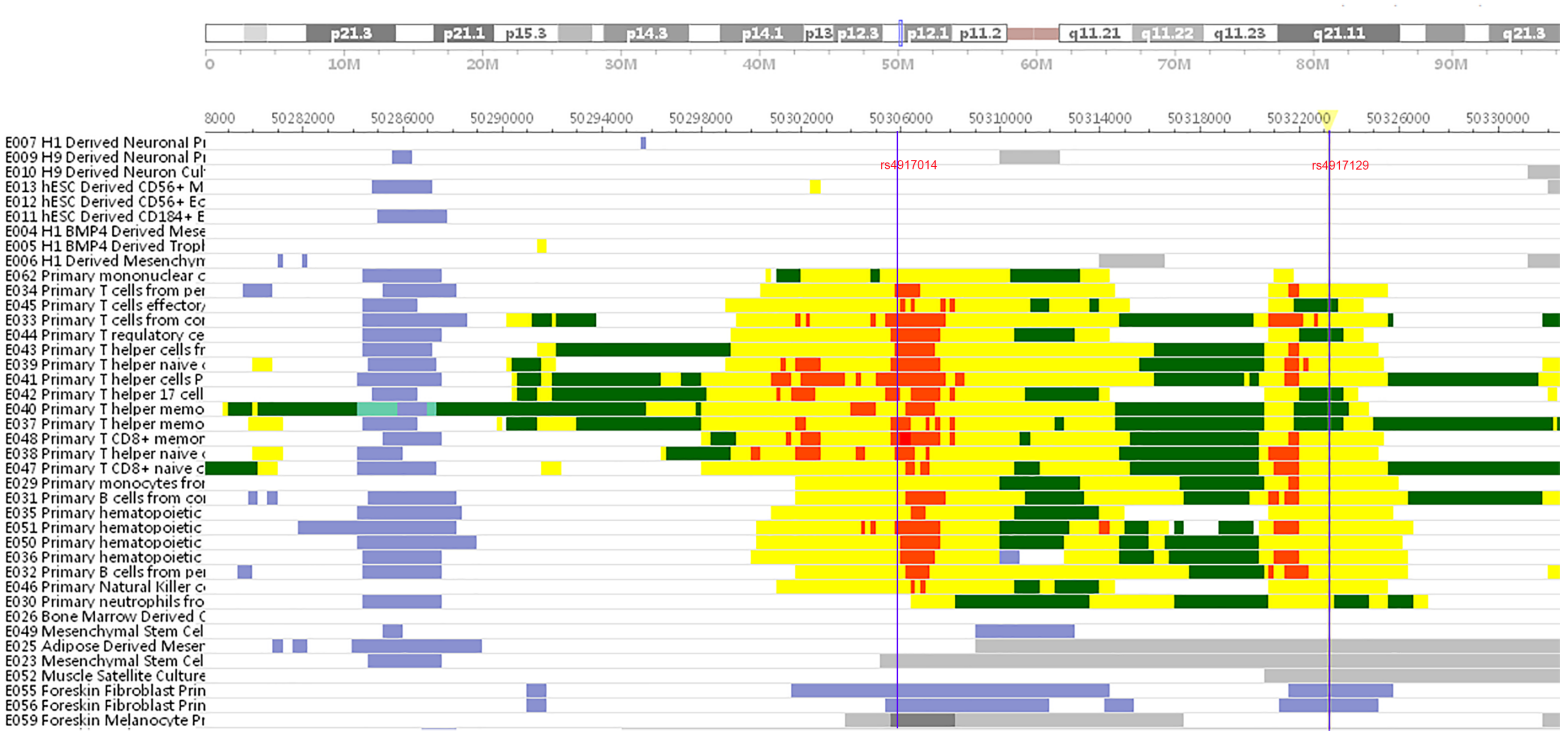
Chromatin states of the rs4917129 locus in different cell lines and tissues. This figure was plotted according to the core-15 states model from the Roadmap Epigenetic project[22]. The majority of samples used in this analysis were immune-related cell lines or tissues, with several other cell lines or tissues as controls. Chromatin states are denoted with different colors; in particular, yellow indicates an enhancer chromatin state.

Because both genotyped SNPs and the imputed SNPs in LD were located in an intergenic region near *IKZF1*, we explored whether these SNPs had potential functional consequences. We found that both rs4917129 and rs4917014 had RegulomeDB scores and were located in putative enhancer regions in some immune-related cell lines (see Fig 2). Moreover, rs4917014 was indicated as an expression quantitative trait locus (eQTL) of *IKZF1* in parietal tissue in rVarBase. These results suggested that the two SNPs might have functional consequences.

**Fig 3:**
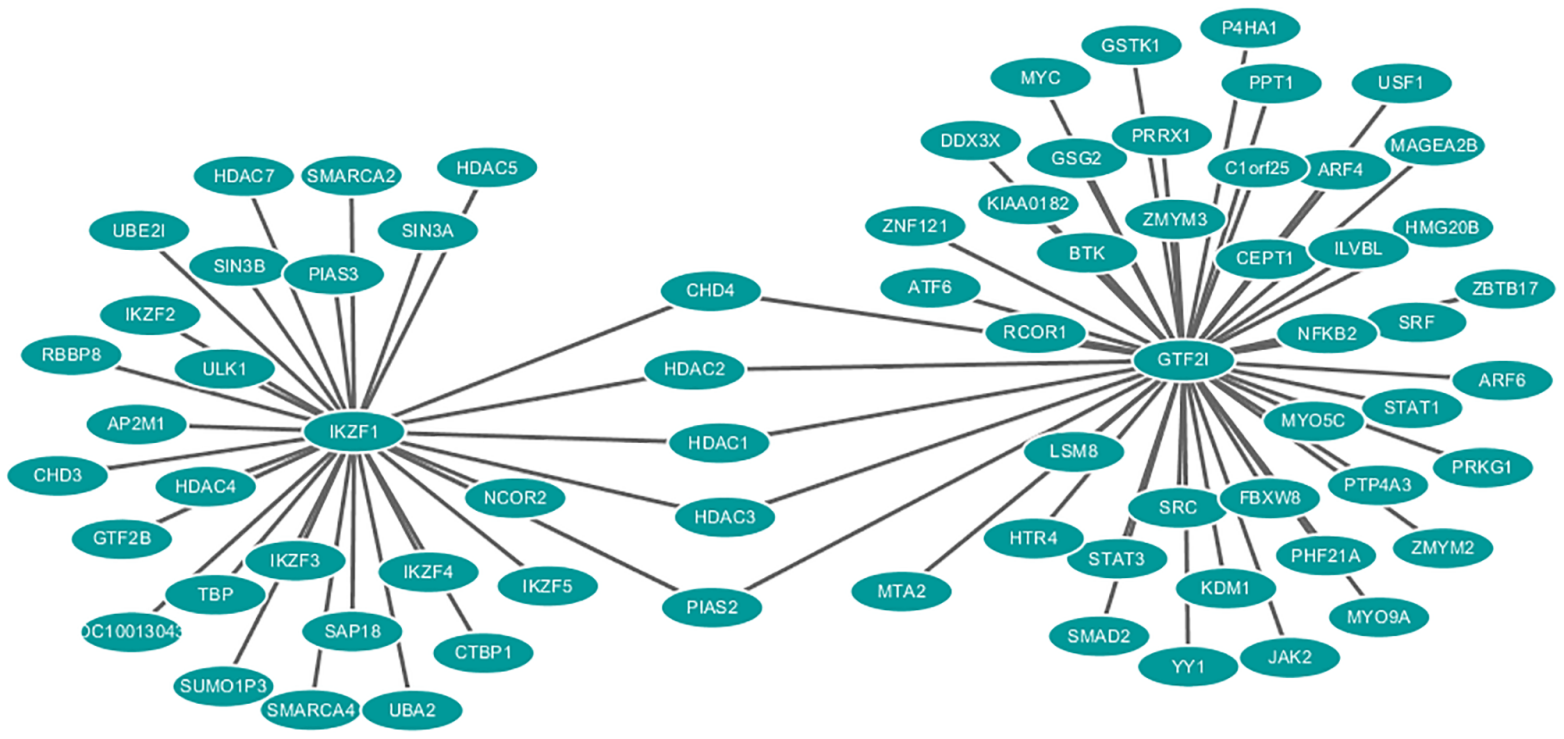
Protein-protein interaction network for *IKZF1*. The edge between the hub and partners indicates the interaction. The networks were drawn using Cytoscape 2.8.2.

To further investigate the possible function of this significant locus in the development of pSS, we performed a PPI network analysis. We did not find any known GWAS-identified candidate genes with direct physical interactions with *IKZF1*. However, *IKZF1* exhibited a common interaction of protein members of histone deacetylase families, e.g., HDAC1, HDAC2 and HDAC3, with our newly identified pSS gene *GTF2I* (general transcription factor IIi)[5] (see Fig 3). *GTF2I* encodes a universal transcription factor IIi (TFII-I), which is a multifunctional phosphoprotein that play roles in transcription and signal transduction. *GTF2I* has been reported to be one of the major genes responsible for neurological deficits in Williams-Beuren syndrome. Numerous studies have shown that TFII-I plays an important role in signal-induced transcriptional regulation in response to various signaling pathways, including immune signaling by both B cells and T cells[23]. Histone deacetylase proteins play a key role in the regulation of eukaryotic gene expression and are related to many complex disorders[24]. Moreover, *HDAC1* was found to be highly expressed in infiltrating lymphocytic foci in the labial glands of patients with SS[25].

## Discussion

Through replication and meta-analyses in Han Chinese subjects, our study identified two SNPs, rs4917129 and rs4917014, near the *IKZF1* locus that were significantly associated with pSS. Among these SNPs, rs4917129 reached the level of genome-wide significance.

Ikaros family zinc finger 1, the protein product encoded by *IKZF1*, is a member of the DNA-binding protein family that functions as a transcription factor in the thymus, spleen, peripheral blood lymphocytes and lymph nodes. Previous studies have reported that Ikaros plays an important role in the development of several lymphocytes, such as T and B cells[26]. It was found that Ikaros is required in the early stages of T and B cell specification during fetal development, and different phases of the development of lymphoid lineages in adults are dependent on the regulation of Ikaros[27]. Moreover, in homozygous mutant mice in which the DNA-binding domain of the *IKZF1* gene was changed, erythroid and myeloid lineages were complete, but T, B and natural killer (NK) cells and their precursors were lacking[28]. Thus, Ikaros is essential for normal lymphocyte occurrence, development and differentiation. Abnormal Ikaros may lead to paralysis of the immune system and further influences the occurrence of autoimmune diseases, such as pSS. Since 1999, *IKZF1* has been implicated in the roles, involved in several hematologic traits or abnormalities, such as erythrocyte measures, SLE, and acute lymphoblastic leukemia (ALL)[8, 29–31]. To the best of our knowledge, our patients with pSS had no history of the above-mentioned diseases.

Furthermore, previous studies have shown that interferon regulatory factors (*IRFs*) (e.g., *IRF-5* and *IRF-8*) regulate the expression of *IKZF1* and Ikaros[32, 33], thereby regulating the induction of inflammatory cytokines and type 1 interferons. Additionally, Ikaros is involved in the regulation of *STAT4* in human T cells[7]. Interestingly, associations of *IRF-5* and *STAT4* with pSS have been confirmed in previous GWAS of pSS[5, 6]. If further experimental evidence confirms our finding that *IKZF1* is associated with pSS, then *IKZF1*, *IRFs* and *STAT4* may be further demonstrated to play a role in disease pathogenesis by regulating immune-related cell activities of some signaling pathways involved in pSS, such as the STAT4 and interferon (IFN) pathways. We also found indirect interactions between *IKZF1* and *GTF2I* through the PPI analysis, and *GTF2I* was significantly associated with pSS in our previous GWAS[5]. These lines of evidence suggest that *IKZF1* may represent a promising pSS candidate gene. Our findings indicate a significant association between *IKZF1* and pSS and provide reliable evidence for the involvement of *IKZF1* in the pathogenesis of pSS.

Rs4917014 and rs4917129 are located in the 5' region of *IKZF1*, and the distance between these two SNPs and *IKZF1* exon 1 is greater than 50 kb. Recent studies on *IKZF1* have suggested that rs4917129 or other polymorphisms in strong linkage equilibrium with this SNP have no influence on *IKZF1* gene expression levels or functional amino acid replacements[34, 35]. Nonetheless, the experimental results of the present study indicate that these two SNPs may regulate *IKZF1* alternative splicing and might alter the efficiency of alternative splicing. Further investigations and analyses are needed to elucidate the role of *IKZF1* in the pathogenesis of pSS and to identify causal variants related to the regulation of alternative splicing. We also used rVarBase, an updated database of regulatory features of human variants to further explore the biological relationship between the two SNPs and pSS. We found that rs4917014 and rs4917129 are located in a putative enhancer region, and their chromatin is in an active state in T lymphocytes, B lymphocytes and hematopoietic stem cells (HSCs). In contrast, in other organs such as the heart, digestive organs, brain and muscle tissue, chromatin states are silent. In addition, rs4917014 is a *cis*-regulatory element for *IKZF1* in parietal tissue. Together, these findings imply an important role in regulating *IKZF1* for these SNPs, which are significantly associated with susceptibility to pSS.

One limitation of our study is that we selected only two tag SNPs at the *IKZF1* locus for genotyping. Other causal variant(s) associated with pSS may exist. Therefore, we need to further explore the relationship between other polymorphisms at the *IKZF1* gene locus and pSS in the Han Chinese population. Additionally, whether *IKZF1* is associated with pSS in other ethnic groups needs to be further verified. In addition, we have presented allele/genotype frequencies in East Asian populations from 1000 Genomes (Phase 3) and our populations (see S2 Table). We analyzed our samples (cases and controls in discovery GWAS), HapMap2 CHB (Northern Han Chinese) and 1000 Genomes Phase1 CHS (Southern Han Chinese) using a principal components analysis (PCA). For eigenvector 3, it has been shown that most of our samples overlapped with most of the CHB samples, while a small proportion of our samples grouped together with a small group of CHB samples and all the CHS samples. Thus, the internal population stratification of Han Chinese is not well described, which may be another limit of our study. However, the results (see S1 Fig) show that our cases and controls were well matched, and the *P*-values were highly similar after the PC1 and PC3 values were added as covariates (for detailed results, see S1 Table). This limit should not impact our results substantially since the cases and controls in our study were well matched. Moreover, no population outliers (> 6 s.d. from the mean for any of the top ten eigenvectors) were present in the GWAS discovery stage, while no outliers could be detected in our replication cohort because only two SNPs were genotyped. This is also a limitation of our study. It is worth mentioning that most pSS-associated genes described to date play important roles in immune function rather than directly encoding salivary or lacrimal components; this is also true of *IKZF1*. A recent review of pSS proposed a potential framework for understanding the roles of these pSS-associated immune genes; it suggested that the risk polymorphisms could enhance the likelihood of autoimmunity, but an individual polymorphism was not sufficiently powerful to directly cause disease[36]. Our results highlight the likelihood that altered immune activity is the major driving factor in pSS pathogenesis. The exact causative agents that trigger this abnormal immune activity remain to be defined.

In summary, this study is the first to report that *IKZF1* is a novel susceptibility locus for pSS in the Chinese Han population.

## Conclusions

This study identified two common variants (rs4917129 and rs4917014) near *IKZF1* that are significantly associated with pSS in Han Chinese. Among them, rs4917129 reached the level of genome-wide significance level. Furthermore, multi-level evidence indicates that *IKZF1* may play a role in disease pathogenesis: the bioinformatics analysis showed that both SNPs are located in a putative enhancer area of immune-related cell lines or tissues, and the PPI analysis revealed that *IKZF1* together with *GTF2I* (an SS susceptibility gene newly identified by GWAS) interacted with histone deacetylase family proteins. These results provide new insights into the pathogenesis of pSS and the first evidence that *IKZF1* is a novel susceptibility locus for pSS in the Chinese Han population.

## Supporting information

S1 Fig Plots of two eigenvectors derived from the principal components analysis (PCA) for the Chinese sub-population in our sample. PCA was performed on our samples (cases and controls in discovery GWAS), HapMap2 CHB (Northern Han Chinese) and 1000 Genomes Phase1 CHS (Southern Han Chinese) using EIGENSOFT 4.2.
(TIFF)

S1 File Supplementary compressed materials for PPI plot. Data files (see Interaction.txt) containing interactions between proteins have been provided in the annex, and the graph for PPI was drawn using Cytoscape 2.8.2 (the relevant file for imaging is also provided, see pSS_PPI.cys).
(RAR)

S1 Table Conditional analysis results for the high-quality (Rsq > 0.5) imputed SNPs with *P*-values < 1E-03. (DOCX)

S2 Table Allele frequencies of the two SNPs in East Asian populations from 1000 Genomes (Phase 3) and our populations. (DOCX)
