# Supplementary figures and images for: Common variants near IKZF1 are associated with primary Sjögren's syndrome in Han Chinese
Source: PLoS One. 2017 May 26;12(5):e0177320. doi: 10.1371/journal.pone.0177320 (PMC5446195; doi:10.1371/journal.pone.0177320)

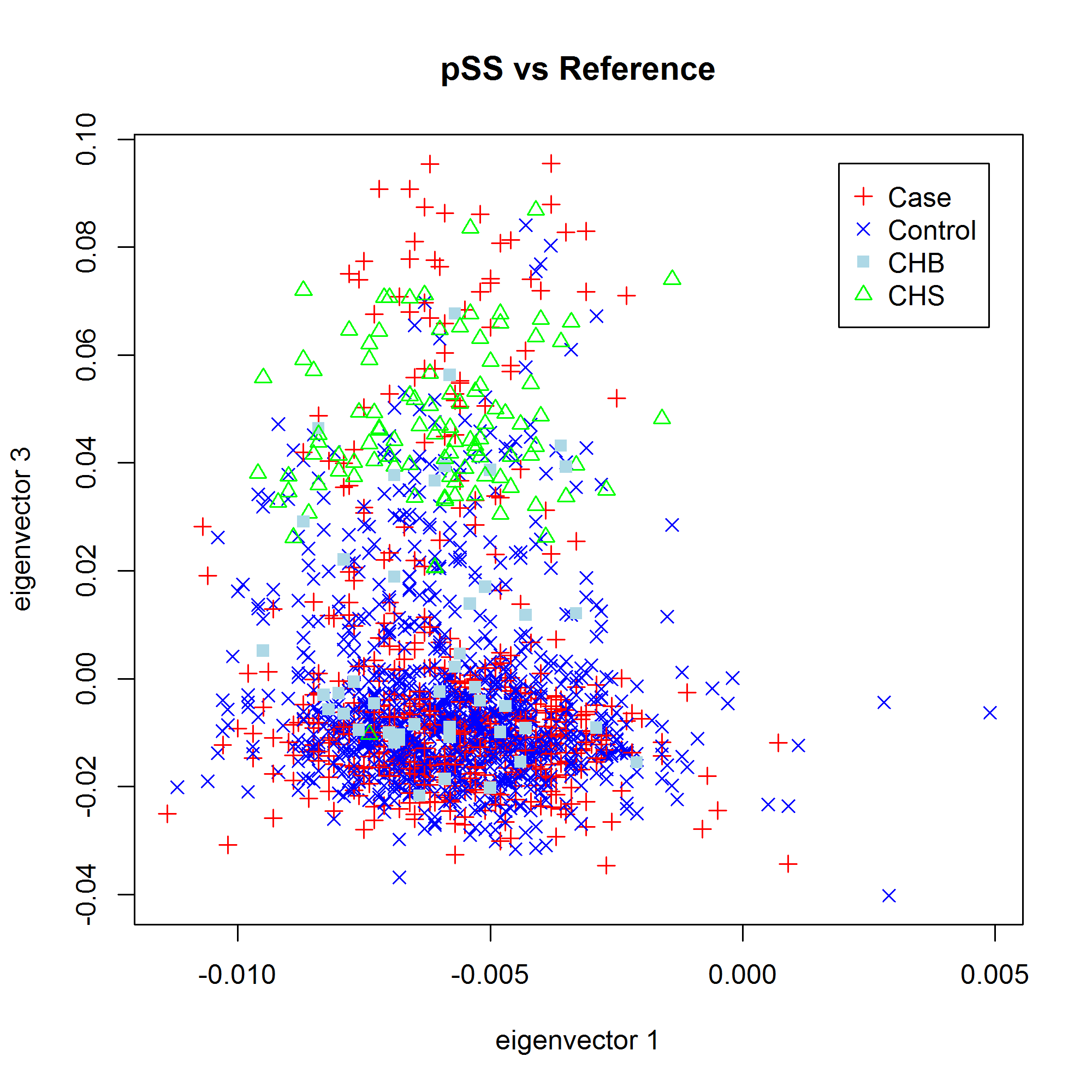

Supplement: S1 Fig — PCA was performed on our samples (cases and controls in discovery GWAS), HapMap2 CHB (Northern Han Chinese) and 1000 Genomes Phase1 CHS (Southern Han Chinese) using EIGENSOFT 4.2. (TIFF) [file pone.0177320.s001.tiff]
