# Supplementary material for: Common variants near IKZF1 are associated with primary Sjögren's syndrome in Han Chinese
Source: PLoS One. 2017 May 26;12(5):e0177320. doi: 10.1371/journal.pone.0177320 (PMC5446195; doi:10.1371/journal.pone.0177320)
Supplement: S1 Table — (DOCX) [file pone.0177320.s003.docx]

**S1 Table.** Conditional analysis results for the high-quality (Rsq > 0.5) imputed SNPs with *P*-values < 1E-03.

| **SNP** | **R^2^_rs4917129_** | **R^2^_rs4917014_** | **R^2^_rs62445350_** | **G/I^a^** | ***P*-value^b^** | ***P_1_*-value ^c^** | ***P_2_*-value ^d^** | ***P_1+2_*-value ^e^** | ***P-*value^g^** | ***P-*value^h^** |
| --- | --- | --- | --- | --- | --- | --- | --- | --- | --- | --- |
| rs62445350 | 0.875 | 0.675 | -- | I | 1.03E-06 | 0.008512 | 0.01326 | 0.04074 | NA | 1.06E-06 |
| rs55935382 | 0.877 | 0.674 | 0.998 | I | 1.17E-06 | 0.01036 | 0.01508 | 0.04891 | NA | 1.21E-06 |
| rs11185602 | 0.877 | 0.674 | 0.998 | I | 1.17E-06 | 0.01036 | 0.01508 | 0.04891 | NA | 1.21E-06 |
| rs876038 | 0.891 | 0.663 | 0.983 | I | 2.76E-06 | 0.03691 | 0.03335 | 0.1475 | NA | 2.87E-06 |
| rs876037 | 0.891 | 0.663 | 0.983 | I | 2.76E-06 | 0.03691 | 0.03335 | 0.1475 | NA | 2.87E-06 |
| rs876039 | 0.891 | 0.663 | 0.983 | I | 2.76E-06 | 0.03691 | 0.03335 | 0.1475 | NA | 2.87E-06 |
| rs62447171 | 0.891 | 0.663 | 0.983 | I | 2.76E-06 | 0.03691 | 0.03335 | 0.1475 | NA | 2.87E-06 |
| rs17552904 | 0.891 | 0.663 | 0.983 | I | 2.76E-06 | 0.03691 | 0.03335 | 0.1475 | NA | 2.87E-06 |
| rs4598207 | 0.795 | 0.593 | 0.905 | I | 3.28E-06 | 0.05868 | 0.0305 | 0.149 | 0.9969 | 3.29E-06 |
| rs34767118 | 0.802 | 0.609 | 0.917 | I | 4.80E-06 | 0.08776 | 0.04524 | 0.2278 | 0.7034 | 4.82E-06 |
| rs11773763 | 0.802 | 0.609 | 0.917 | I | 4.80E-06 | 0.08776 | 0.04524 | 0.2278 | 0.7034 | 4.82E-06 |
| rs4917014 | 0.573 | -- | 0.675 | G | 1.43E-05 | 0.09357 | NA | NA | 0.5918 | 1.48E-05 |
| rs4917015 | 1 | 0.573 | 0.875 | I | 1.81E-05 | NA | 0.1091 | NA | 0.4009 | 1.81E-05 |
| rs1026422 | 1 | 0.573 | 0.875 | I | 1.81E-05 | NA | 0.1091 | NA | 0.4009 | 1.81E-05 |
| rs17634369 | 1 | 0.573 | 0.875 | I | 1.81E-05 | NA | 0.1091 | NA | 0.4009 | 1.81E-05 |
| rs4917129 | -- | 0.573 | 0.875 | G | 1.81E-05 | NA | 0.1091 | NA | 0.4009 | 1.81E-05 |
| rs6964608 | 1 | 0.573 | 0.875 | I | 1.81E-05 | NA | 0.1091 | NA | 0.4009 | 1.81E-05 |
| rs11185603^f^ | 0.578 | 0.994 | 0.68 | I | 2.67E-05 | 0.1472 | NA | NA | 0.7863 | 2.77E-05 |
| rs876036 | 0.762 | 0.551 | 0.848 | I | 4.29E-05 | 0.494 | 0.1849 | 0.7865 | 0.2286 | 4.46E-05 |
| rs1870027 | 0.686 | 0.485 | 0.777 | I | 5.60E-05 | 0.4113 | 0.1501 | 0.5883 | 0.501 | 5.60E-05 |
| rs4385425 | 0.504 | 0.878 | 0.601 | I | 0.000186 | 0.3496 | 0.3233 | 0.3003 | 0.8639 | 0.0001924 |
| rs2715094 | 0.004 | 0.014 | 0.002 | I | 0.0009879 | 0.002235 | 0.004475 | 0.003752 | 0.001931 | 0.000953 |

NA indicates no result after analysis.

^a^G indicates genotyped; I indicates imputed.

^b^*P*-value after imputation and without conditional analysis.

^c^*P*-value after adding the allelic dosage for rs4917129 as a covariate.

^d^*P*-value with adding the allelic dosage for rs4917014 as a covariate.

^e^*P*-value with adding the allelic dosage for both rs4917014 and rs4917129 as covariates.

^f^Reported to be associated with systemic lupus erythematosus (SLE) in a 2016 study (PMID: 26502338).

^g^*P*-value after adding only the allelic dosage for rs62445350 as a covariate.

^h^*P*-value after imputation, without conditional analysis and after adjusting for PC1 and PC3.

Each association test was conducted based on an additive logistic regression model.
