# Supplementary material for: Common variants near IKZF1 are associated with primary Sjögren's syndrome in Han Chinese
Source: PLoS One. 2017 May 26;12(5):e0177320. doi: 10.1371/journal.pone.0177320 (PMC5446195; doi:10.1371/journal.pone.0177320)
Supplement: S2 Table — (DOCX) [file pone.0177320.s004.docx]

**S2 Table.** Allele frequencies of the two SNPs in East Asian populations from 1000 Genomes (Phase 3) and our populations.

| **SNP** | **Sample** | | **Allele frequencies** | |
| --- | --- | --- | --- | --- |
|  |  |  |  |  |
| **rs4917129** | **1000 Genomes, Phase 3** |  | **T** | **C** |
|  |  | CHB | 0.7136 | 0.2864 |
|  |  | CHS | 0.6429 | 0.3571 |
|  |  | JPT | 0.5433 | 0.4567 |
|  | **Pre-GWAS^a^** | Control | 0.7405 | 0.2595 |
|  |  | Case | 0.8090 | 0.1910 |
|  | **Current study** | Control | 0.7491 | 0.2509 |
|  |  | Case | 0.7925 | 0.2075 |
| **rs4917014** | **1000 Genomes, Phase 3** |  | **T** | **G** |
|  |  | CHB | 0.6650 | 0.3350 |
|  |  | CHS | 0.6000 | 0.4000 |
|  |  | JPT | 0.5192 | 0.4808 |
|  | **Pre-GWAS^a^** | Control | 0.6839 | 0.3161 |
|  |  | Case | 0.7569 | 0.2431 |
|  | **Current study** | Control | 0.6955 | 0.3045 |
|  |  | Case | 0.7391 | 0.2609 |

CHB, Northern Han Chinese. CHS, Southern Han Chinese. JPT, Japanese in Tokyo, Japan.

^a^Pre-GWAS indicates previous GWAS sample.
